# Coronavirus Disease: A Review of a New Threat to Public Health

**DOI:** 10.7759/cureus.7276

**Published:** 2020-03-15

**Authors:** Kamleshun Ramphul, Stephanie G Mejias

**Affiliations:** 1 Pediatrics, Shanghai Jiao Tong University School of Medicine/Shanghai Xin Hua Hospital, Shanghai, CHN; 2 Family Medicine/Pediatrics/Internal Medicine, The University Iberoamericana Unibe School of Medicine/Robert Reid Cabral Children's Hospital, Santo Domingo, DOM

**Keywords:** coronavirus disease, covid-19, severe acute respiratory syndrome coronavirus 2, sars-cov-2, wuhan

## Abstract

In December 2019, several patients from Wuhan, China were admitted to hospitals with symptoms of pneumonia. As the number of patients presenting with similar symptoms started to rise, the causative agent was eventually isolated from samples. It was initially called the 2019 novel coronavirus (2019-nCoV) and has been recently relabelled as severe acute respiratory syndrome coronavirus 2 (SARS-CoV-2); the disease it causes has been named coronavirus disease 2019 (COVID-19). Over the next few weeks, the virus spread from Wuhan to affect different provinces in China and, after a few months, it is now present in 109 countries. As of March 10, 2020, there have been 113,702 confirmed cases globally, and 4,012 deaths have been registered. The World Health Organization (WHO) called COVID-19 a pandemic on March 11, 2020. There are multiple drug trials going on with some positive results. However, since no vaccine is available, the best way to combat the virus is by preventive methods.

## Introduction and background

Over the last few decades, the world has seen the existence of new viruses that posed serious threats to global health. In late December 2019, several patients in Wuhan, China started reporting symptoms that resembled pneumonia. A new virus was identified and initially called the 2019 novel coronavirus (2019-nCoV). The World Health Organization (WHO) eventually changed the name of the virus to severe acute respiratory syndrome coronavirus 2 (SARS-CoV-2) [1]. The disease it causes has been named coronavirus disease 2019 (COVID-19). The SARS-CoV is a positive-stranded RNA virus that originates from the Coronaviridae family. Other viruses from the same family include the severe acute respiratory syndrome coronavirus (SARS-CoV), which appeared in 2002, and Middle East respiratory syndrome coronavirus (MERS-CoV), which was reported in 2012 [2]. Since the virus is spreading worldwide, on March 11, 2020, the WHO officially described the COVID-19 outbreak as a pandemic.

## Review

Epidemiology

As of March 10, 2020, the WHO has reported that there are 113,702 confirmed cases globally, and 4012 deaths have been registered; 71% of all confirmed cases (80,924) and 78% of all deaths related to COVID-19 (3140) are from China and its territories. Since the first reported case in Wuhan, 109 other countries have declared that they have at least one confirmed case of COVID-19. The WHO has officially classified China as a “very high risk” region for COVID-19 [3].

Mainland China and territories

The province of Hubei, which includes Wuhan, currently has the highest confirmed number of cases and deaths in mainland China (67,760 cases and 3,024 deaths). Among the regions in China that have crossed the four-digit mark, Guangdong, Henan, Zhejiang, and Hunan each have 1,353, 1,272, 1,215, and 1,018 cases, respectively (Table 1). The second-highest number of deaths was reported in Guangdong with 22 cases.

**Table 1:** Number of confirmed cases and deaths linked with COVID-19 in China as of March 10, 2020 (reported by the WHO) SAR: special administrative region; WHO: World Health Organization

Province	Confirmed cases	Deaths
Hubei	67,760	3,024
Guangdong	1,353	8
Henan	1,272	22
Zhejiang	1,215	1
Hunan	1,018	4
Anhui	990	6
Jiangxi	935	1
Shandong	758	6
Jiangsu	631	0
Chongqing	576	6
Sichuan	539	3
Heilongjiang	481	13
Beijing	429	8
Shanghai	342	3
Hebei	318	6
Fujian	296	1
Guangxi	252	2
Shaanxi	245	1
Yunnan	174	2
Hainan	168	6
Guizhou	146	2
Tianjin	136	3
Shanxi	133	0
Liaoning	125	1
Gansu	124	2
Hong Kong SAR	115	3
Jilin	93	1
Xinjiang	76	3
Ningxia	75	0
Inner Mongolia	75	1
Taipei and environs	45	1
Qinghai	18	0
Macao SAR	10	0
Xizang	1	0
Total	80,924	3,140

Outside China

While South Korea and Iran were initially the two most affected countries outside China, the number of cases started to rise in Italy in late February and, as of March 10, 2020, Italy is the country outside China with the highest number of total confirmed cases (9,172) (Table 2).

**Table 2:** Number of confirmed cases and deaths related to COVID-19 outside of China as of March 10, 2020 (reported by the WHO) WHO: World Health Organization

Country	Number of confirmed cases	Deaths
South Korea	7,513	54
Japan	514	9
Italy	9,172	463
France	1,402	30
Germany	1,138	2
Spain	1,024	28
Iran	7,161	237
USA	472	19
Other countries (excluding China)	3,686	23

What is severe acute respiratory syndrome coronavirus 2?

SARS-CoV-2 is a positive-sense, single-stranded RNA virus. The SARS-CoV-2 virion is about 50-200 nm in diameter and consists of four main structural proteins; spike (S), envelope (E), membrane (M), and nucleocapsid (N) [4]. The S protein allows the virus to bind to the host’s cell membrane. The angiotensin-converting enzyme 2 (ACE2) receptors on host cells have been found to be the target of S proteins. It then undergoes structural changes to fuse with the host, and this eventually allows viral genes to enter the host cell [5-7].

Genomic comparison has shown that the SARS-CoV-2 has an 80% resemblance to Rhinolophus sinicus bat and 96% resemblance with the Rhinolophus affinis bat [8,9]. One research team found that one sample of the virus had a 99% genomic similarity with pangolins and suggested that the animal may be an intermediate host to the virus [10,11].

According to the Centers for Disease Control and Prevention (CDC), the transmission of SARS-CoV-2 occurs mostly person-to-person via respiratory droplets within a range of 180 cm. The virus can also be transmitted if a person touches a mucosal surface after touching an object with the virus on it [12].

What are the clinical symptoms of COVID-19?

While the incubation period of the virus was initially thought to be 14 days, multiple cases have been reported with shorter timelines. A study by Guan et al. calculated the median incubation period to be four days with a lower interquartile range of two days and an upper interquartile range of seven days. In their study, the most common finding on imaging was ground-glass opacity on CT (56.4%) [13]. They found that 43.8% had a fever on admission and 88.7% during hospitalization. The cough was also a common symptom and was seen in 67.8% of patients. The Chinese Center for Disease Control and Prevention has reported that 87% of confirmed cases were in adults aged between 30 and 79 years. The mortality and case fatality rate increased with increasing ages; the case fatality rate was 8% in patients aged between 70 and 79 years while it was 15% in those aged 80 years or more [14].

A study involving 10 children showed that most presented with fever (80%) while 60% had cough [15]. All children presented with mild symptoms and they all recovered. The study also reported that the patients had prolonged virus shedding in the respiratory tract and feces, even during their convalescent stage. A second study involved nine infected infants aged 1-11 months. Four out of the nine patients presented with fever; one infant had no symptoms but was tested positive for the virus. None of the infants needed mechanical ventilation or had to be admitted in intensive care [16].

How are patients tested for SARS-CoV-2?

Samples from the upper respiratory tract are used to test for the virus. Polymerase chain reaction (PCR) is used to identify their viral RNA. If the test is positive, the diagnosis of SARS-CoV-2 is confirmed. Negative tests with a strong suspicion, such as clinical symptoms or exposure, can be repeated using samples from other respiratory sites [17,18].

Treatment of COVID-19

Multiple antiviral regimens are being tried to help patients with severe symptoms of the virus. Lopinavir and ritonavir have been used in some clinical trials. Lim et al. reported that the drugs helped their patient as he improved clinically and viral loads decreased significantly [19]. Four COVID-19 patients were recruited by Wang et al. for a study in Shanghai, China, and they also improved with a combined therapy of lopinavir and ritonavir [20]. In Singapore, confirmed cases that were hospitalized were also given the combined antiviral therapy of lopinavir and ritonavir. While some patients reported improvement in their symptoms, four patients developed nausea, vomiting, or diarrhea, and three patients showed elevated liver function test results [21].

Several other treatment options such as Remdesivir (Gilead Sciences, Foster City, CA), peptide (EK1), neuraminidase inhibitors, chloroquine, and arbidol have also been suggested [22]. There are multiple research teams trying to investigate a possible vaccine for the virus. The role of the spike protein in the viral infectivity and pathogenesis is a possible preventive target [23].

How to prevent COVID-19?

The CDC recommends multiple steps to prevent the transmission and risk of SARS-CoV-2. Frequent hand washing lasting at least 20 seconds by using soap and water is advised. Hand sanitizers with at least 60% alcohol can also be used as an alternative. The public has also been told to avoid touching mucosal surfaces such as the mouth and the nose with hands that have not been washed. Anyone showing symptoms of the virus should try to seek appropriate medical help. They should also limit their exposure to other unaffected people and cover their noses and mouths when coughing or sneezing. They are also advised to wear a facemask if they present with symptoms. Frequent disinfection and cleaning are advised for groups that are at risk of contracting the virus [24].

## Conclusions

The SARS-CoV-2 is spreading across the world at an alarming rate. The elderly and immunocompromised patients are most vulnerable to the mortal repercussions of the virus. While some treatment protocols have shown some promise, there is at present no confirmed cure for the virus and no vaccine has been developed. With proper preventive measures, the virus can be contained and the population protected.

## References

[1] (2020). Centers for Disease Control and Prevention: coronavirus disease 2019 (COVID-19) - situation summary. https://www.cdc.gov/coronavirus/2019-ncov/summary.html.

[2] (2020). Centers for Disease Control and Prevention: human coronavirus types. https://www.cdc.gov/coronavirus/types.html.

[3] (2020). WHO: coronavirus disease (COVID-2019) situation reports. https://www.who.int/emergencies/diseases/novel-coronavirus-2019/situation-reports.

[4] Chen N, Zhou M, Dong X (2020). Epidemiological and clinical characteristics of 99 cases of 2019 novel coronavirus pneumonia in Wuhan, China: a descriptive study. Lancet.

[5] Letko M, Marzi A, Munster V (2020). Functional assessment of cell entry and receptor usage for SARS-CoV-2 and other lineage B betacoronaviruses. Nat Microbiol.

[6] Gralinski LE, Menachery VD (2020). Return of the Coronavirus: 2019-nCoV. Viruses.

[7] Lu R, Zhao X, Li J (2020). Genomic characterisation and epidemiology of 2019 novel coronavirus: implications for virus origins and receptor binding. Lancet.

[8] Zhou P, Yang XL, Wang XG (2020). A pneumonia outbreak associated with a new coronavirus of probable bat origin. Nature.

[9] Wong MC, Cregeen SJ, Ajami NJ, Petrosino J (2020). Evidence of recombination in coronaviruses implicating pangolin origins of nCoV-2019. BioRxiv.

[10] Peng X, Xu X, Li Y, Cheng L, Zhou X, Ren B (2020). Transmission routes of 2019-nCoV and controls in dental practice. Int J Oral Sci.

[11] Wahba L, Jain N, Fire AZ, Shoura MJ, Artiles KL, McCoy MJ, Jeong DE (2020). Identification of a pangolin niche for a 2019-nCoV-like coronavirus through an extensive meta-metagenomic search. BioRxiv.

[12] (2020). Centers for Disease Control and Prevention: how COVID-19 spreads. https://www.cdc.gov/coronavirus/2019-ncov/about/transmission.html.

[13] Guan WJ, Ni ZY, Hu Y (2020). Clinical characteristics of coronavirus disease 2019 in China. N Engl J Med.

[14] Wu Z, McGoogan JM (2020). Characteristics of and important lessons from the coronavirus disease 2019 (COVID-19) outbreak in China: summary of a report of 72314 cases from the Chinese Center for Disease Control and Prevention. JAMA.

[15] Cai J, Xu J, Lin D (2020). A case series of children with 2019 novel coronavirus infection: clinical and epidemiological features. Clin Infect Dis.

[16] Wei M, Yuan J, Liu Y, Fu T, Yu X, Zhang ZJ (2020). Novel coronavirus infection in hospitalized infants under 1 year of age in China. JAMA.

[17] (2020). Centers for Disease Control and Prevention: persons evaluated for 2019 novel coronavirus — United States, January 2020. https://www.cdc.gov/mmwr/volumes/69/wr/mm6906e1.htm?s_cid=mm6906e1_x.

[18] (2020). Coronavirus disease 2019 (COVID-19). https://www.uptodate.com/contents/coronavirus-disease-2019-covid-19#H2325386707.

[19] Lim J, Jeon S, Shin HY (2020). Case of the index patient who caused tertiary transmission of COVID-19 infection in Korea: the application of lopinavir/ritonavir for the treatment of COVID-19 infected pneumonia monitored by quantitative RT-PCR. J Korean Med Sci.

[20] Wang Z, Chen X, Lu Y, Chen F, Zhang W (2020). Clinical characteristics and therapeutic procedure for four cases with 2019 novel coronavirus pneumonia receiving combined Chinese and Western medicine treatment. Biosci Trends.

[21] Young BE, Ong SWX, Kalimuddin S (2020). Epidemiologic features and clinical course of patients infected with SARS-CoV-2 in Singapore. JAMA.

[22] Lu H (2020). Drug treatment options for the 2019-new coronavirus (2019-nCoV). Biosci Trends.

[23] (2020). National Institutes of Health: novel coronavirus structure reveals targets for vaccines and treatments. https://www.nih.gov/news-events/nih-research-matters/novel-coronavirus-structure-reveals-targets-vaccines-treatments.

[24] (2020). Centers for Disease Control and Prevention: coronavirus disease 2019 (COVID-19) - how to protect yourself. https://www.cdc.gov/coronavirus/2019-ncov/about/prevention.html.

